# Peripapillary microvascular changes in patients with systemic hypertension: An optical coherence tomography angiography study

**DOI:** 10.1038/s41598-020-63603-6

**Published:** 2020-04-16

**Authors:** Yong-Il Shin, Ki-Yup Nam, Woo-Hyuk Lee, Cheon-Kuk Ryu, Hyung-Bin Lim, Young-Joon Jo, Jung-Yeul Kim

**Affiliations:** 1 Department of Ophthalmology, Chungnam National University College of Medicine, Daejeon, Republic of Korea; 2 Department of Ophthalmology, Gyeongsang National University Changwon Hospital, Changwon, Republic of Korea

**Keywords:** Optic nerve diseases, Retinal diseases

## Abstract

The purpose of this study was to investigate changes in peripapillary microvasculature using optical coherence tomography angiography (OCTA) in systemic hypertension (HTN) patients. This was a cross-sectional study. Based on the duration of HTN, seventy-eight HTN patients were divided into two groups. (HTN group 1: <10 years, 38 eyes; HTN group 2: ≥10 years, 40 eyes) and 90 control subjects. All subjects underwent 6 × 6 mm OCTA scan centered on the optic nerve head. We analyzed peripapillary vessel density (VD) and perfusion density (PD) in superficial capillary plexus among three groups. The average ganglion cell-inner plexiform layer (GC-IPL) and retinal nerve fiber layer (RNFL) thicknesses of HTN group 2 were thinner than those of the control group (p = 0.016, and 0.035, respectively). HTN group 2 showed lower peripapillary VD and PD than the control group. However, there were no differences between HTN group 1 and the control group in OCT and peripapillary OCTA parameters. In HTN patients, the peripapillary VD, PD and GC-IPL, RNFL thicknesses correlated significantly. OCTA showed that the peripapillary VD and PD were lower in HTN patients with a duration ≥10 years compared with those of normal controls. Peripapillary microvasculature was correlated with the RNFL and GC-IPL thicknesses. HTN duration should therefore be considered when evaluating peripapillary microvasculature using OCTA.

## Introduction

Cardiovascular disease is one of the more common causes of death in developing country, and hypertension (HTN) is the most common treatable risk factor. According to a US study, approximately 68 million (31%) of patients ≥18 years of age are reported to have HTN^1^, and approximately one billion worldwide are affected by HTN^2^. With advances in medical technology, the life expectancy has been extended, and the number of patients with HTN has increased.

Uncontrolled HTN can cause vascular changes in many organ systems, such as the brain, heart, kidneys, and eyes, due to elevated arterial pressure and increased peripheral resistance. Vascular changes in the eye can be directly visualized using funduscopy. Arteriolar narrowing is a hallmark of hypertensive retinopathy (HTNR), and the narrowing being characterized as either focal or diffuse. This occurs when the systemic blood pressure rises to maintain constant blood flow by autoregulation of the retinal circulation. The destruction of the inner blood-retinal barrier and vascular endothelial structure can then lead to cotton wool spots, retinal hemorrhage, and intraretinal lipid deposits.

Funduscopy, optical coherence tomography (OCT), and fluorescein angiography (FA) may be helpful in the diagnosis and evaluation of retinal pathological changes in HTN. In our previous OCT study, the ganglion cell-inner plexiform layer (GC-IPL) and peripapillary retinal nerve fiber layer (RNFL) thicknesses were lower in chronic HTN and relieved HTNR patients than normal controls^3,4^, Furthermore, in previous optical coherence tomography angiography **(**OCTA) studies, foveal microvascular perfusion was decreased, which correlated with GC-IPL thinning in patients with HTN^5,6^.

We assumed that RNFL thinning in patients with HTN should be related with peripapillary microvascular changes. We therefore analyzed changes in peripapillary microvasculature in patients with HTN using OCTA, and identified the associations between OCT and peripapillary OCTA parameters.

## Methods

### Subjects

This retrospective, cross-sectional study was approved by the institutional review board of Chungnam National University Hospital, and was conducted to the tenets of the Declaration of Helsinki. The requirement for obtaining informed patient consent was waived by the institutional review board of Chungnam National University Hospital due to the retrospective nature of the study. The medical records at the Chungnam National University Hospital inclusive were reviewed to identify HTN patients who visited retina clinic.

Diabetic patients were excluded because diabetes significantly affects the inner retinal layer and microvasculature. HTN was diagnosed as systolic blood pressure ≥140 mmHg or diastolic blood pressure ≥90 mmHg^7^ at Chungnam National University Hospital. They underwent regular follow-up for blood pressure control, and all participants were well-controlled blood pressure for at least the past year. The participants were divided into three groups: control group (negative history of systemic disease and no ocular disease, 90 eyes), HTN group 1 (patients with HTN < 10 years, 38 eyes), and HTN group 2 (patients with HTN ≥ 10 years, 40 eyes). We excluded HTNR using fundus photography of patients in the HTN groups. One eye from each participant was included. Only one eye was randomly selected if both eyes were eligible. In the power calculation using G*Power 3.1^8^, the power of the average peripapillary VD and PD were 0.970 and 0.967, respectively.

The exclusion criteria were systemic diseases other than HTN and hyperlipidemia, glaucoma, optic nerve disorders, a best-corrected visual acuity (BCVA) < 20/25, intraocular pressure (IOP) > 21 mmHg, high myopia [axial length (AL) ≥ 26.0 mm or spherical equivalent (SE) > −6 diopters], and a history of intraocular surgery.

All patients underwent slit-lamp biomicroscopy, BCVA, SE, IOP, and AL using an IOL master, fundus photography, dilated fundus examination, OCT, and OCTA.

### Optical coherence tomography (OCT) and optical coherence tomography angiography (OCTA) measurement

OCT examination was performed using a spectral domain OCT (Cirrus HD-OCT; Carl Zeiss Meditec, Dublin, CA, USA). The skilled examiner performed all imaging. Central macular thickness (CMT) and GC-IPL thickness were analyzed using a macular cube scan. Average RNFL thickness was analyzed using an optic disc cube scan. We excluded with signal strength (SS) less than 7 or poor centration or segmentation error in OCT scans.

OCTA imaging was acquired using the Zeiss Cirrus HD-OCT with Angioplex, with a light source at 840 nm and an A-scan rate of 68 KHz. We used only scans without segmentation errors and motion artifacts and with SS greater than 9. The superficial capillary plexus (SCP) images were segmented with an internal limiting membrane and the inner plexiform layer and the deep capillary plexus (DCP) was segmented with an inner nuclear layer and the outer plexiform layer.

**Figure 1:**
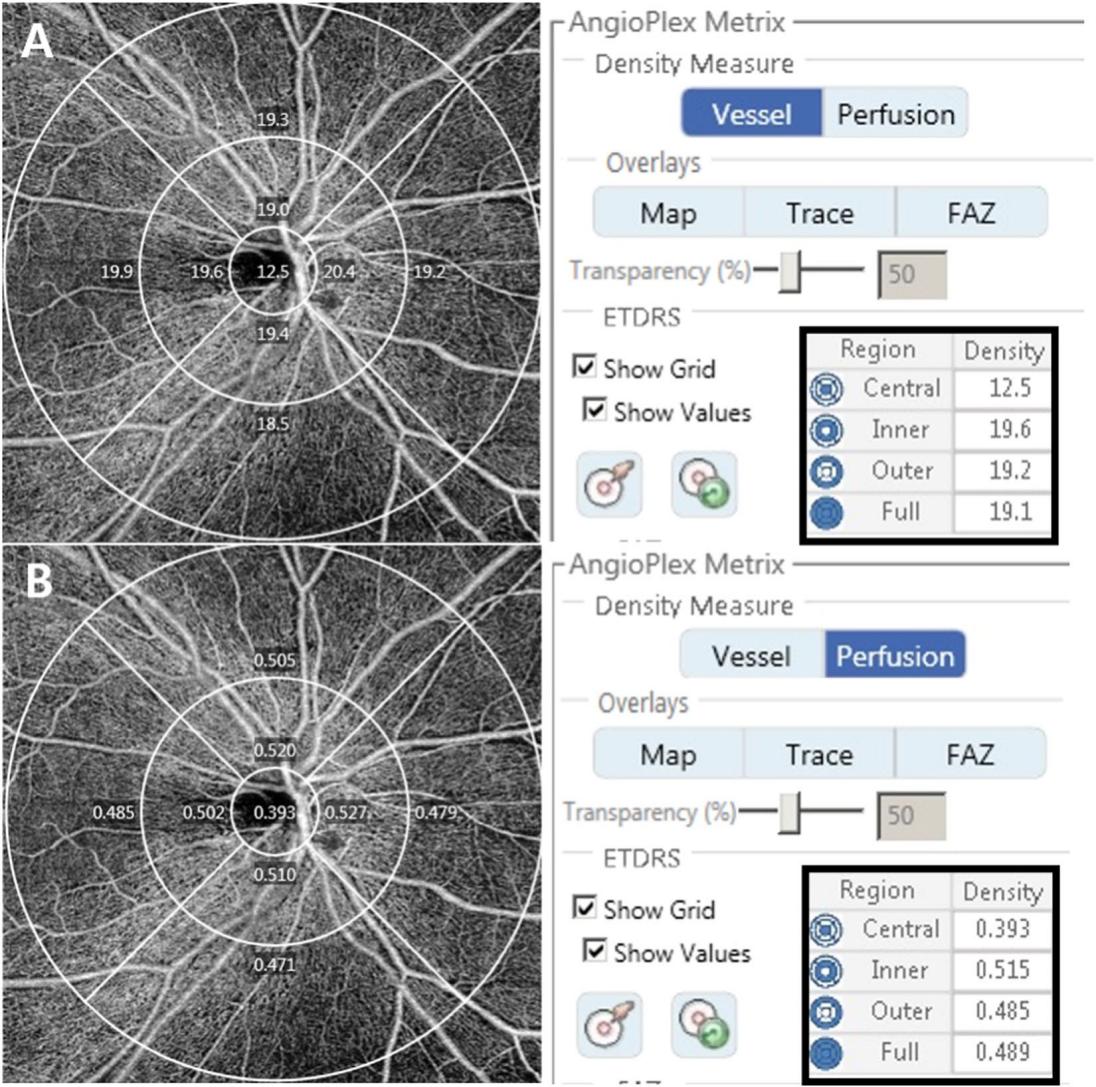
Optical coherence tomography angiography 6 × 6 mm scan image centered on the optic disc. The en face image of the superficial layer overlaid with the Early Treatment of Diabetic Retinopathy Study grid. The diameters of the three concentric circles are 1, 3, and 6 mm. The measurement tool (AngioPlex software, version 10.0; Carl Zeiss Meditec) provided (**A**) peripapillary vessel density and (**B**) perfusion density measurements in individual subfields. The bold box shows the automatic quantitative measurements for an average of the inner ring, outer ring, and full area.

The optic disc centered 6 × 6 mm OCTA scans were acquired to evaluate peripapillary microvasculature. A 6 × 6 mm scans were acquired containing 350 × 350 scans each, and B-scans were repeated twice at each cross-section. Vessel density (VD) and perfusion density (PD) are defined as follows; VD means total length of perfused vasculature per unit area in a region of measurement and PD means the total area of perfused vasculature per unit area in a region of measurement. The AngioPlex software automatically displays the measured value of the SCP according to the subfield of the ETDRS. We only studied SCP because the built-in software provided automatically quantified values of SCP only. We analyzed the peripapillary VD and PD of the average and quadrants of the inner ring, outer ring, and full area (Fig. 1). We included with SS more than 9 and without segmentation errors and motion artifacts in OCTA images.

### Statistical analysis

Statistical analysis were performed with SPSS version 22.0 (Chicago, IL, USA). For statistical analyses, BCVA values were transformed to the logarithm of the minimum angle of resolution (log MAR) values. One-way ANOVA with Bonferroni’s post-hoc test and the chi-squared test were applied to compare the clinical characteristics, OCT, and OCTA parameters among groups. To determine the relationship between peripapillary OCTA and OCT parameters, Pearson’s correlation was used. P < 0.05 was considered statistically significant.

## Results

### Patient characteristics

After excluding poor quality OCTA images (6 eyes of HTN group 1, 7 eyes of HTN group 2, and 6 eyes of normal controls), Seventy-eight eyes from HTN patients (38 in HTN group 1 and 40 in HTN group 2) and 90 control eyes were enrolled in the study. The durations of HTN were 4.2 ± 2.4 years in HTN group 1 and 16.3 ± 5.8 years in HTN group 2 (*p* < 0.001). There were no significant difference among the three groups in age, sex, laterality, BCVA, SE, IOP, AL, and SS of OCTA images (Table 1).

**Table 1:** Demographics and clinical features of the study subjects.

	Control group (n = 90)	HTN group 1 (n = 38)	HTN group 2 (n = 40)	p-value
Age (years)	60.1 ± 8.9	60.6 ± 10.1	62.8 ± 8.4	0.292^*^
Sex (male/female)	38/52	22/16	15/25	0.154^†^
Laterality (od/os)	44/46	17/21	20/20	0.882^†^
Duration of hypertension (years)		4.2 ± 2.4	16.3 ± 5.8	**<0.001**^**‡**^
Systolic blood pressure (mmHg)	115.6 ± 9.7	119.1 ± 8.3	117.1 ± 8.8	0.148^*^
Diastolic blood pressure (mmHg)	79.8 ± 7.6	82.2 ± 5.3	81.1 ± 6.3	0.182^*^
BCVA (log MAR)	−0.02 ± 0.08	−0.01 ± 0.07	0.02 ± 0.08	0.123^*^
Spherical equivalent (diopters)	−0.39 ± 2.06	0.07 ± 1.24	0.01 ± 1.43	0.289^*^
Intraocular pressure (mmHg)	14.3 ± 2.9	15.2 ± 2.6	15.2 ± 3.0	0.118^*^
Axial length (mm)	23.71 ± 0.96	23.85 ± 0.84	23.62 ± 0.89	0.542^*^
Signal strength	9.7 ± 0.5	9.6 ± 0.5	9.6 ± 0.5	0.571^*^

HTN = hypertension; BCVA = best-corrected visual acuity; log MAR = logarithm of the minimum angle of resolution.

Values are presented as mean ± standard deviation unless otherwise indicated.

^*^p-value for one-way analysis of variance.

^†^p-value for chi-square test.

^‡^p-value for Student’s *t*-test (HTN group 1 vs. 2).

### OCT measurements

The CMT did not show a difference among the three groups (*p* = 0.135). The GC-IPL and peripapillary RNFL thicknesses showed statistically significant differences in the three groups (*p* = 0.019, and 0.037, respectively). Using Bonferroni’s test, the GC-IPL and RNFL thicknesses in HTN group 2 (80.2 ± 6.2 and 92.2 ± 8.1 μm, respectively) were lower than those of the control group (83.3 ± 5.8 and 96.0 ± 7.7 μm, respectively). However, there were no differences between HTN group 1 and the control group (Table 2).

**Table 2:** Comparison of the central macular thickness, average GC-IPL thickness, and RNFL thickness among groups.

	Control group (n = 90)	HTN group 1 (n = 38)	HTN group 2 (n = 40)	p-value^*^	p-value^†^	p-value^‡^	p-value^§^
Central macular thickness (μm)	251.7 ± 18.4	249.0 ± 23.5	244.0 ± 20.7	0.135			
Average GC-IPL thickness (μm)	83.3 ± 5.8	81.9 ± 5.2	80.2 ± 6.2	**0.019**	0.633	**0.016**	0.588
Average RNFL thickness (μm)	96.0 ± 7.7	94.1 ± 8.5	92.2 ± 8.1	**0.037**	0.675	**0.035**	0.824

HTN = hypertension; GC-IPL = ganglion cell-inner plexiform layer; RNFL = retinal nerve fiber layer.

Values are presented as mean ± standard deviation.

^*^The p-value was obtained using one-way analysis of variance.

^†^The p-value was obtained using post hoc tests (Bonferroni) between the normal control group and HTN group 1.

^‡^The p-value was obtained using post hoc tests (Bonferroni) between the normal control group and HTN group 2.

^§^The p-value was obtained using post hoc tests (Bonferroni) between HTN group 1 and group 2.

### Peripapillary OCTA measurements

The mean values ± standard deviation of OCTA measurements are listed in Table 3 (VD) and Table 4 (PD). The average peripapillary VD and PD of the inner ring, outer ring, and full area were significantly different among the three groups. Using Bonferroni’s test, HTN group 2 showed a lower peripapillary VD and PD than the control group. The average of the full areas for the VD and PD and the average PD of the outer ring were lower in HTN group 2 than in HTN group 1.

**Table 3:** Comparison of superficial peripapillary vessel density among groups.

	Control group (n = 90)	HTN group 1 (n = 38)	HTN group 2 (n = 40)	p-value^*^	p-value^†^	p-value^‡^	p-value^§^
Full area	18.23 ± 0.58	18.02 ± 0.77	17.62 ± 0.72	**<0.001**	0.282	**<0.001**	**0.026**
**Inner ring**							
Average	17.50 ± 1.23	17.12 ± 1.80	16.83 ± 1.32	**0.036**	0.489	**0.039**	1.000
Superior	17.79 ± 1.36	17.58 ± 1.85	17.55 ± 1.61	0.632			
Nasal	18.08 ± 1.41	17.61 ± 2.15	17.54 ± 1.53	0.136			
Inferior	18.26 ± 1.16	18.06 ± 1.30	17.46 ± 1.34	**0.004**	1.000	**0.003**	0.103
Temporal	16.32 ± 2.41	15.26 ± 3.02	15.13 ± 2.54	**0.021**	0.109	**0.049**	1.000
**Outer ring**							
Average	18.90 ± 0.59	18.74 ± 0.63	18.53 ± 1.06	**0.035**	0.872	**0.031**	0.613
Superior	19.19 ± 0.61	19.15 ± 0.68	19.06 ± 1.03	0.622			
Nasal	17.50 ± 1.38	17.47 ± 1.07	17.24 ± 1.65	0.595			
Inferior	18.98 ± 0.78	18.91 ± 0.86	18.88 ± 1.14	0.808			
Temporal	19.87 ± 0.91	19.43 ± 1.31	18.97 ± 1.71	**0.001**	0.193	**0.001**	0.318

HTN = Hypertension.

Values are presented as mean ± standard deviation.

^*^The p-value was obtained using one-way analysis of variance.

^†^The p-value was obtained using post hoc tests (Bonferroni) between the normal control group and HTN group 1.

^‡^The p-value was obtained using post hoc tests (Bonferroni) between the normal control group and HTN group 2.

^§^The p-value was obtained using post hoc tests (Bonferroni) between HTN group 1 and group 2.

**Table 4:** Comparison of superficial peripapillary perfusion density among groups.

	Control group (n = 90)	HTN group 1 (n = 38)	HTN group 2 (n = 40)	p-value^*^	p-value^†^	p-value^‡^	p-value^§^
Full area	0.465 ± 0.013	0.460 ± 0.020	0.449 ± 0.020	**<0.001**	0.274	**<0.001**	**0.019**
**Inner ring**							
Average	0.459 ± 0.032	0.450 ± 0.047	0.441 ± 0.039	**0.049**	0.703	**0.049**	0.934
Superior	0.472 ± 0.041	0.466 ± 0.049	0.468 ± 0.048	0.745			
Nasal	0.482 ± 0.033	0.468 ± 0.061	0.467 ± 0.047	0.118			
Inferior	0.486 ± 0.030	0.485 ± 0.035	0.470 ± 0.041	**0.049**	1.000	0.056	0.159
Temporal	0.393 ± 0.060	0.375 ± 0.077	0.358 ± 0.071	**0.025**	0.794	**0.021**	0.548
**Outer ring**							
Average	0.479 ± 0.014	0.475 ± 0.020	0.465 ± 0.024	**<0.001**	0.843	**<0.001**	**0.038**
Superior	0.496 ± 0.016	0.493 ± 0.017	0.492 ± 0.017	0.310			
Nasal	0.451 ± 0.032	0.449 ± 0.043	0.432 ± 0.047	**0.040**	1.000	**0.040**	0.178
Inferior	0.488 ± 0.019	0.481 ± 0.024	0.476 ± 0.031	**0.036**	0.504	**0.038**	1.000
Temporal	0.481 ± 0.020	0.473 ± 0.024	0.459 ± 0.048	**0.001**	0.443	**<0.001**	0.135

HTN = Hypertension.

Values are presented as mean ± standard deviation.

^*^The p-value was obtained using one-way analysis of variance.

^†^The p-value was obtained using post hoc tests (Bonferroni) between the normal control group and HTN group 1.

^‡^The p-value was obtained using post hoc tests (Bonferroni) between the normal control group and HTN group 2.

^§^The p-value was obtained using post hoc tests (Bonferroni) between HTN group 1 and group 2.

### Association of clinical and OCT parameters with peripapillary OCTA parameters in HTN patients

**Figure 2:**
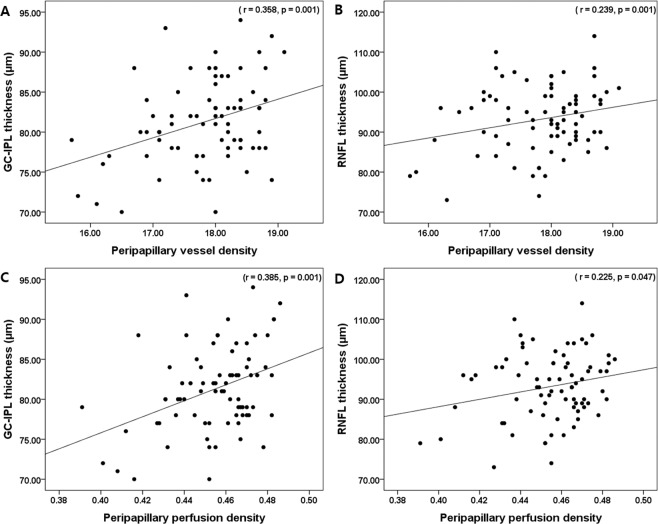
Scatter plots of association with peripapillary OCTA parameters with OCT parameters. Peripapillary vessel density (VD) and perfusion density (PD) were significantly correlated with average ganglion cell-inner plexiform layer (GC-IPL) and retinal nerve fiber layer (RNFL) thickness in HTN patients. Correlation coefficients (r) and *p-*values are shown.

The average GC-IPL and peripapillary RNFL thicknesses were correlated with the peripapillary VD (r = 0.358, *p* = 0.001 and r = 0.239, *p* = 0.035, respectively) and PD (r = 0.385, *p* = 0.001 and r = 0.225, *p* = 0.047, respectively) in HTN patients (Fig. 2). The duration of HTN was not correlated with GC-IPL and RNFL thicknesses, but negatively correlated with the peripapillary VD and PD (r = −0.219, *p* = 0.049 and r = −0.240, *p* = 0.035, respectively). However, age, female sex, BCVA, SE, IOP, AL, mean arterial pressure and CMT were not correlated with the average peripapillary VD and PD. Multivariate regression analysis showed that clinical factors such as age, IOP, sex, BCVA, SE, and mean arterial pressure were not significant factors affecting the average peripapillary VD and PD (see Supplementary Table 1). Age, axial length, and signal strength showed no statistically significant correlation with the average peripapillary VD and PD in normal controls.

## Discussion

Systemic HTN affects the structure and function of blood vessels, and HTN changes are systemic risk factors for cardiovascular disease^9,10^, stroke^11^, and chronic kidney disease^12,13^. In eyes, HTN aggravates diabetic retinopathy^14^, increases the risk of retinal vascular occlusive disease^15,16^, and is associated with ischemic optic neuropathy.^17^ In addition, HTN is associated with ocular diseases such as progression of glaucoma^18,19^, and increases in choroidal neovascularization (CNV) in the fellow eye of patients with one eye affected with CNV^20^.

HTNR is one of the markers of target organ damage in the initial evaluation of HTN patients^21^, and the grade of HTNR is associated with mortality^9,22^. thus, evaluation of retinal changes in HTN patients is clinically important.

Elevated blood pressure results in various changes to the retinal vasculature. These changes are classified as arteriolar changes (arteriolar narrowing, arteriolar wall opacification and arteriovenous nicking) and retinal lesions (retinal hemorrhage, microaneurysm, cotton wool spots, and hard exudate) caused by blood-retinal barrier damage. Hayreh reported that hypertensive changes in the eye affect not only the retina but also the choroid and optic disc^23^.

Funduscopy has been used to diagnose HTNR. However, if there is only arteriolar narrowing during an early stage, it may be difficult to detect these changes using only a fundus examination. Several studies have therefore been conducted to quantitate and differentiate these changes. Fundus photography has been used to measure decreases in retinal arteriolar diameter and the arteriole-to-venule ratio, and has revealed such decreases in high blood pressure patients when compared with normal subjects^24–26^.

OCT is widely utilized for the diagnosis and treatment in retinal diseases and glaucoma. It can distinguish ten retinal layers with high resolution, and has the advantage of quantitatively evaluating the thickness of each layer. In HTN patients without retinopathy or glaucoma, thinning of the GC-IPL and peripapillary RNFL was reported^3,4,27^. In our study, there was no difference in the CMT, but the GC-IPL and RNFL thicknesses were different among the three groups, and those of HTN group 2 was thinner than the control group. In an animal study using rhesus monkeys, the RNFL thickness decreased with chronic arterial hypertension^28^. In addition, previous studies reported that the inner retina during ischemia is more vulnerable to hypoxia, which is consistent with our previous research^29,30^. This reduction in inner retinal thickness may be associated with hypertensive retinal ischemia, such that chronic microvascular disorder is associated with slow inner retinal thinning. Similar to diabetic retinal neurodegeneration, it is possible that hypertensive changes can occur in patients without retinopathy. These were not observed using funduscopy, but there was still the possibility of microvascular damage. However, studies using OCT provide limited information on retinal capillary circulation.

The recently developed technique OCTA has no side effects from contrast agents, in contrast to FA, and has the advantage of being able to measure blood flow and the vascular density in SCP and DCP, separately. There have been many reports on the quantitative analysis of retinal vascular changes and ischemia in diabetic retinopathy, as well as on retinal venous occlusion and glaucoma. Recent studies with OCTA have shown decreased foveal microvascular parameters, increased foveal avascular zone areas in patients with chronic HTN, and relived HTNR. Because of the association between the microvasculature and inner retinal layer thickness, inner retinal thinning may be associated with retinal microcirculation^5,6^. In addition, patients with poorly controlled blood pressure showed a greater decrease in the retinal capillary density compared with patients whose blood pressure is well controlled^31^.

To our knowledge, there has been no previous study on changes to the peripapillary microvasculature according to HTN using OCTA. We found that the average peripapillary VD and PD of the inner ring, outer ring, and full area were different among the three groups. The peripapillary VD and PD of HTN Group 2 were lower than those of normal controls. We evaluated associations between OCT and peripapillary OCTA parameters in HTN patients. The peripapillary VD and PD showed a correlation with the GC-IPL and RNFL thicknesses. The decreased GC-IPL and peripapillary RNFL thickness might be associated with decreased peripapillary microvascular circulation. In the present study, the HTN duration did not correlate with the GC-IPL and RNFL thicknesses, but was correlated with the peripapillary VD and PD. It is presumed that the peripapillary microvasculature was more sensitive to the duration of HTN than the inner retinal thickness.

This study has some limitations. First, in this retrospective, cross-sectional study, it was difficult to identify the occurrence of previous hypertensive events. In general, if there is no acute rise in blood pressure, visual symptoms do not occur, and ophthalmic examinations are often not performed. We excluded patients in HTNR with cotton wool spots, retinal hemorrhage, and severe arteriolar narrowing, thereby minimizing the effect of these on peripapillary OCTA parameters. Second, anti-hypertensive medications and kidney functioning (low estimated glomerular filtration rate levels) could affect the retinal microcirculation^32,33^. Because of the cross-sectional study, we could not investigate these parameters in detail. Previous studies^32,33^ used scanning laser Doppler Flowmetry and fundus photography, so additional studies with OCTA may be needed. Third, a further longitudinal study of temporal sequencing between decreased GC-IPL and RNFL thicknesses and retinal microvasculature should be conducted, and the DCP and choroid investigated, as they may be affected by HTN. Finally, we only analyzed SCP, not DCP and choriocapillaries. However, previous studies have shown that projection artifacts may affect quantitative analysis of DCP and choriocapillaries, and Enders *et al*.^34^ reported that 75% of deep retina images were shown projection artifacts. Further studies on the changes of DCP and choriocapillaries using projection-resolved OCTA will be needed.

Our study excluded systemic diseases other than HTN. It is important to investigate the only effects of HTN on peripapillary microvasculature. In addition, we studied changes in peripapillary optic nerve head perfusion in HTN patients for the first time. Overall, the strength of this study was its characterization of the relationship between peripapillary microvasculature and the RNFL and GC-IPL thicknesses.

In conclusion, this study showed that the peripapillary VD and PD were lower in HTN patients with a duration of ≥10 years, and the peripapillary VD and PD showed a correlation with RNFL and GC-IPL thicknesses. Based on the findings in this study, the effects of HTN duration should be taken into account when evaluating the peripapillary microvasculature using OCTA.

## Supplementary information

Supplementary Table 1.

## Data Availability

Data supporting the findings of the current study are available from the corresponding author on reasonable request.
