# Supplementary material for: Peripapillary microvascular changes in patients with systemic hypertension: An optical coherence tomography angiography study
Source: Sci Rep. 2020 Apr 16;10:6541. doi: 10.1038/s41598-020-63603-6 (PMC7162882; doi:10.1038/s41598-020-63603-6)
Supplement: Supplementary file 1 — Supplementary Table 1. [file 41598_2020_63603_MOESM1_ESM.doc]

**Supplementary information**

**Peripapillary microvascular changes in patients with systemic hypertension: An optical coherence tomography angiography study**

Yong-Il Shin, MD^1^, Ki Yup Nam, MD, Ph.D^2^, Woo Hyuk Lee, MD^1^,

Cheon Kuk Ryu, MD^1^, Hyung-Bin Lim, MD, Ph.D^1^, Young-Joon Jo, MD, Ph.D^1^,

Jung-Yeul Kim, MD, Ph.D^1^

Department of Ophthalmology, Chungnam National University College of Medicine, Daejeon, Republic of Korea^1^

Department of Ophthalmology, Gyeongsang National University Changwon Hospital, Changwon, Republic of Korea^2^

**Supplementary Table 1.** Multivariate regression for determining the factors associated with average peripapillary vessel density and perfusion density.

|  | Peripapillary vessel density | |  | Peripapillary perfusion density | |
| --- | --- | --- | --- | --- | --- |
|  | β (95% CI) | p-value |  | β (95% CI) | p-value |
| Age (years) | -0.016 (-0.036, 0.004) | 0.117 |  | 0.000 (-0.001, 0.000) | 0.244 |
| Intraocular pressure (mmHg) | -0.033 (-0.094, 0.029) | 0.290 |  | 0.000 (-0.002, 0.001) | 0.707 |
| Female sex | -0.005 (-0.378, -0.368) | 0.979 |  | -0.001 (-0.011, 0.008) | 0.763 |
| BCVA (LogMAR) | -1.018 (-3.383, 1.346) | 0.393 |  | -0.033 (-0.096, 0.029) | 0.290 |
| Spherical equivalent (diopter) | -0.080 (-0.225, 0.065) | 0.275 |  | -0.002 (-0.006, 0.002) | 0.305 |
| Mean arterial pressure (mmHg) | -0.006 (-0.029, 0.017) | 0.614 |  | 0.000 (-0.001, 0.000) | 0.647 |

β = regression coefficients; CI = confidential interval; BCVA = best-corrected visual acuity; LogMAR = logarithm of the minimum angle of resolution
